# Is Follow-up Co-Morbidity Assessment via Laboratory Investigations in Older High Energy Trauma Patients Justified? - A Prospective-Retrospective Study

**DOI:** 10.5704/MOJ.2303.001

**Published:** 2023-03

**Authors:** G Jain, G Vadivelu, A Krishna, R Malhotra, V Sharma, K Farooque

**Affiliations:** 1 Department of Orthopaedics, All India Institute of Medical Sciences, New Delhi, India; 2 Department of Orthopaedics, SGT Medical College Hospital and Research Institute, Gurugram, India

**Keywords:** fracture, osteoporotic, high energy, laboratory diagnosis

## Abstract

**Introduction:**

The objective of the current study was to test our hypothesis that older patients sustaining high energy trauma need to be evaluated for their comorbidities similar to geriatric patients sustaining low energy trauma.

**Materials and methods:**

This study was a retrospective-prospective analysis of 173 patients of more than 50 years of age enrolled between November 2017 and December 2018. Herewith, we have compared retrospectively collected laboratory investigations of 124 fragility fracture patients with prospectively collected laboratory investigations of 49 patients with high energy trauma. The laboratory investigations, including the liver function tests, renal function tests, indices of calcium metabolism, serum electrolytes, complete blood counts, and bone mineral density (BMD) scores.

**Results:**

Both groups were similar to each other as far as baseline demographic characteristics were concerned. The proportion of female patients and patients with non-osteoporotic range BMD (T-score >-2.5) was significantly higher in the high-energy fracture group (P value <0.05). Hypoalbuminemia (<3.4gm/dl) 17.3%, abnormalities sodium (<135mmol/L or >148mmol/L) 23.2%, Anaemia (<10g/dl) 12.7%, Hypercalcemia (>10.4mg/dl) 16.3%, Vitamin D deficiency (<20ng/ml) 17.3% are the common laboratory abnormality found in study population. No statistically significant difference was found among the two groups in terms of laboratory investigation abnormalities.

**Conclusion:**

The laboratory investigation abnormality in an older patient with a clinical fracture is independent of the mechanism of injury. The results of the current study emphasise the need for a comprehensive laboratory workup in older patients with either high- energy fractures or fragility fractures.

## Introduction

As population is ageing in most parts of the world, and people are staying active until an older age, the number of elderly patients requiring trauma care is growing^1^. As per estimates, geriatric patients with a fracture will represent 40% of all trauma patients by 2050^2^. Among the geriatric patients with a fracture one-fourth suffer from high-energy injuries, while rest present with a fragility fracture^3^.

Older patients with a fracture require a special care, as compared to the younger population they sustain a more severe injury, require prolonged hospitalisation, and have a higher mortality rate^1^. There exists a different treatment protocol in literature for older patients with low and high energy fractures. In addition to fracture treatment, patients with fragility fractures also undergo extensive laboratory analysis to diagnose any pre-existing comorbidities during their follow-up visit in the Fracture Liaison Service (FLS) clinic.

The FLS is a multidisciplinary dedicated approach to manage fragility fractures^4^. A subclinical pre-existing medical condition compromising fracture healing can be present in more than a quarter of fragility fracture patients^4-9^. This approach of care has not only reduced the possibilities of secondary fractures but also have improved the quality of life and survival rates^10^.

However, in the high energy group, comorbidities often go undiagnosed and untreated since there is no specific protocol available in the literature for managing comorbidities in them like a dedicated fracture liaison service. The present study was thus conducted with this intent in mind, to bring to the fore that high energy group of patients also require a specific protocol on the lines of fracture liaison service for detection of their comorbidities similar to the patients in the low energy group. The objective of the current study was to test our hypothesis that older patients sustaining high energy trauma will have altered laboratory parameters as frequently as the patients with fragility fractures of the same age group. Therefore, patients with high energy trauma need to be evaluated for their comorbidities, similar to geriatric patients sustaining low energy trauma.

## Materials and Methods

The present study was a retrospective-prospective analysis of 173 patients more than 50 years of age. Herewith, we have compared retrospectively collected data of 124 fragility fracture patients with prospectively collected data of 49 patients with high energy trauma. It was conducted at a tertiary level teaching institute. Institutional ethical committee approval was obtained before conducting the study (IECPG-139/23.04.2020). Written consent was obtained from the patients included prospectively in the study, while retrospective data was obtained from the hospital records after ethical clearance.

Low energy trauma was defined as falls from standing height or a lesser injury. In the low energy group (group-L), we included patients treated in our hospital’s fracture liaison service from November 2017 to December 2018. Whereas, in the high energy group, we prospectively added patients older than 50 years of age who suffered a more severe injury such as after falling from stairs, roadside accident, pedestrian injury, assault, fall from a bicycle or being hit by an animal. Patients with trauma to more than one body region, and those who required intensive care at any stage of the treatment, were excluded from the study. We had included fragility fracture patients older than fifty years of age in our FLS clinic as advised in most guidelines^6-9^. To make both the groups comparable, we made 50 years as the yardstick for older age even in the high energy group (group-H). All patients qualifying the inclusion criteria presenting to our hospital during the study period were included in the study.

Patients’ laboratory workup were the primary outcome measures. It included liver function tests, renal function tests, calcium and bone metabolism indices, serum electrolytes and complete blood count. Parameters mentioned in the present study were primarily used to detect underlying comorbidity of the patient. The authors considered it prudent to omit investigations like CPK and ABG from the battery of investigations as these parameters, besides being out of line with the objective of the study, also add to the overall cost of the lab analysis. Trauma or any surgical procedure performed can alter the outcome of laboratory investigations like renal and liver function tests and CBC^11^. We used to conduct all the investigations two weeks after any surgical or non-surgical modality to treat the fracture in the low energy group in our FLS. The rationale behind this strategy was to allow a sufficient buffer period for the blood parameters to return to their original baseline values, thereby enabling the authors to evaluate the baseline blood profile picture of the two groups for comorbidity analysis. We followed the same strategy for the high energy group. We have not analysed the groups based on the known comorbidities as we want to analyse the use of extensive laboratory analysis irrespective of the known comorbidities.

We determined the normal range of each test based on the standards set by our clinical laboratory. We interpreted a haemoglobin level less than 10gm/dl as moderate or severe anaemia and a 25(OH)Vitamin D level of less than 20ng/ml as Vitamin D deficiency. Patients with an estimated glomerular filtration rate based on the serum creatinine (eGFRcr) less than 60mL/min/1.73m^2^ were regarded to have a renal compromise. It was calculated using the Chronic Kidney Disease Epidemiology Collaboration (CKD-EPI) 2009 equation^12^. We staged the chronic kidney disease (CKD) as per the kidney disease outcomes quality initiative committee criteria^13^.

In addition, we collected the data related to the patient’s age, sex, body mass index, and bone mineral density (BMD) from the records of our FLS clinic. We obtained these variables of patients with high energy fractures during the follow-up evaluation at two weeks. Due to recumbency, we could not measure the height and weight of some patients, while some data was also missing from our FLS records.

The BMD estimate, which was the secondary outcome variable, was determined using a Hologic discovery dual-energy X-ray absorptiometry (DXA) system. We calculated the T-score utilising Hologic data for white females. The use of reference data meant for white females may lead to over-diagnosis of osteoporosis in Asians due to their smaller size^14,15^. However, there is a lack of prospective data regarding the normal BMD for the Indian population^16^. Thus, the data of white females are used routinely to estimate the T-scores^16^, and this strategy has been found reasonable by previous authors^15^. Implementing the World Health Organisation definition, we classified a T-score less than -2.5 as osteoporosis, between -1 to -2.5 as osteopenia, and higher than -1 as healthy^4^. The T-score was determined using the bone density of the neck of femur (of the uninjured side in case of hip fracture). The DXA outcome of seven patients of group-H was not available as these patients denied to undergo this test.

Statistical analysis: Categorical variables were analysed using the Chi-square test or Fischer’s exact test. Independent t-test or rank-sum test was applied to compare quantitative variables expressed as mean ± standard deviation. A p-value of less than 0.05 was considered statistically significant. We checked whether selected crucial laboratory outcomes, like hyperparathyroidism, vitamin D deficiency, anaemia, and others, were more frequently encountered among patients with a history of fragility fracture (dependent variable) using the logistic regression analysis. To examine the ability of the number of positive such parameters to separate the patients into those with or without fragility fracture, we constructed a receiver operating characteristic (ROC) curve. The authors used the statistical software Stata 14.0 to conduct the data analysis.

## Results

**Fig. 1:**
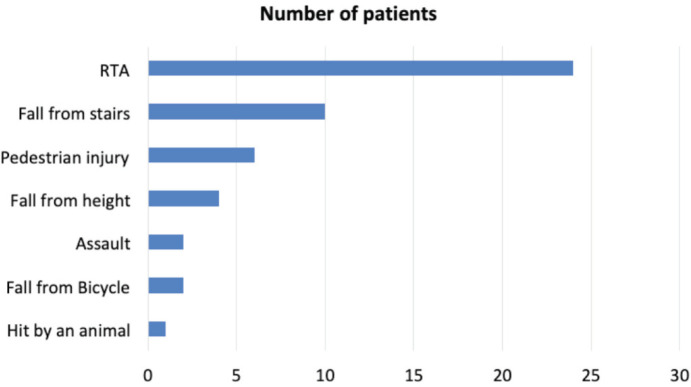
Chart showing the frequency of different modes of injury in patients with high energy fracture. (RTA: road traffic accident).

A road traffic accident was the most common mode of injury among patients with high energy fracture (Fig. 1). Distal radius fracture was the most common fracture in the low-energy group, while intertrochanteric fracture was the most common fracture in group-H (Table I).

**Table I:** Comparison of fracture distribution in patients with high energy and low energy injury. Five patients in group-H and two patients in group-L had fracture at two sites

Fracture Zone	Group-H		Group-L		P-Value
	Number	Percentage	Number	Percentage	
Pertrochantric	13	24.1	29	23	0.85
Distal radius	7	13	44	34.9	0.0035
Proximal humerus	6	11.1	10	7.9	0.57
Vertebral body	5	9.3	9	7.1	0.76
Proximal tibia	4	7.4	1	0.8	0.03
Metatarsal	3	5.6	5	4	0.7
Both bone leg	3	5.6	1	0.8	0.08
Neck of femur	2	3.7	8	6.3	0.73
Shaft of femur	2	3.7	3	2.4	0.64
Metacarpal	2	3.7	0	0	-
Distal tibia	2	3.7	0	0	-
Malleolar	1	1.9	3	2.4	1.00
Distal ulna	1	1.9	1	0.8	0.51
Distal femur	1	1.9	1	0.8	0.51
Forearm	1	1.9	1	0.8	0.51
Distal phalanx	1	1.9	0	0	-
Acetabulum	0	0	3	2.4	-
Metacarpal	0	0	2	1.6	-
Clavivle	0	0	2	1.6	-
Intercondylar humerus	0	0	1	0.8	-
Patella	0	0	1	0.8	-
Humerus shaft	0	0	1	0.8	-

To recognise any confounding bias generated because of the effect of known and unknown factors in the study, both the high energy and low energy group patients were compared for typical confounding variables as discussed below. Patients of both groups belong to the same age bracket of more than 50 years reporting a clinical fracture in our hospital. Both the groups were similar in terms of demographic characteristics like age, height, weight, and BMI (Table II). However, the proportion of females was significantly higher in group-H (P=0.04). Compared to group-H, patients in group-L were significantly more osteoporotic with a lower mean T-score (P=0.03), and more number of osteoporotic patients (PR, 2.1; 95 % CI, 1.0–4.3).

**Table II:** Comparison of demographic parameters and risk factors among patients with high energy injury (group-H) with that of patients with fragility fracture (group-L)

Variables	Group-L		Group-H		PR (95% CI)	P-value
	n (%) / Mean (SD)	N	n (%) / Mean (SD)	N		
Age > 70 years	25 (20.2)	124	12 (24.5)	49	0.8 (0.4- 1.7)	0.53
Female gender	85 (68.5)	124	41 (83.7)	49	0.4 (0.2- 1.0)	0.04
T- score < -2.5	70 (56.5)	124	16 (38.1)	42	2.1 (1.0- 4.3)	0.04
Age	64.0 (9.5)	124	63.4 (8.9)	49	-	0.73
Weight	58.3 (13.2)	110	59.9 (10.5)	35	-	0.50
Height*	153.6 (7.3)	108	152.8 (6.8)	35	-	0.55
BMI	24.6 (4.9)	108	25.9(5.5)	35	-	0.2
T SCORE	-2.6 (1)	124	-2.2 (1.2)	42	-	0.03

Abbreviations - N: Number of patients whose data were available, n: number of patients qualifying the criteria. BMI: Body mass index, CI: Confidence interval, PR: Prevalence ratio, SD: Standard deviation. *Height was measured in centimetres.

**Fig. 2:**
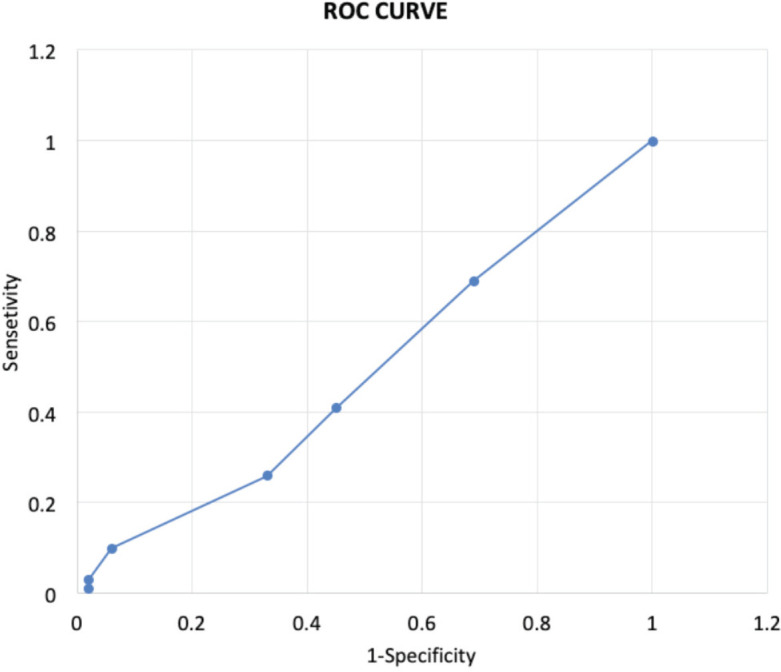
Receiver operating characteristic (ROC) curve: the ROC curve is passing closer the diagonal line, and the area under the curve is 0.57.

There was no statistically significant difference in the mean value of any laboratory criteria between two groups (Table III). Furthermore, as estimated using unadjusted logistic regression analyses, the probability of crucial laboratory outcomes being abnormal was not significantly different in both groups (Table IV). The receiver operating characteristics curve was very close to the diagonal line, i.e. the no-discrimination line and the area under the curve was 0.57, which signify that the number of positive parameters selected for regression analysis has failed to discriminate patients of two groups (Fig. 2).

**Table III:** Laboratory variables of the study subjects in patients with fragility fracture (group-L) and patients with a high-energy fracture (group-H)

Sl. No.	Variable	Normal Range	Group-L (n=124)		Group-H (n=49)		P-value
			Mean (SD)	N	Mean (SD)	N	
1	Haemoglobin	14.0-17.5 g/dL	12.2 (1.9)	120	11.7 (1.5)	37	0.07
2	TLC	4.5-11.0 × 109/L	8.3 (2.0)	120	8.2 (2.1)	37	0.6
3	Neutrophils	48 % to 77%	63.0 (8.2)	115	64.0 (9.1)	35	0.5
4	Eosinophils	0.3 % to 7%	3.3 (2.3)	115	3.1 (2.0)	35	0.7
5	Lymphocytes	10 % to 40%	30.0 (7.8)	114	29.4 (7.6)	35	0.7
6	Monocytes	0.6 % to 9.6%	3.7 (2.2)	116	3.3 (2.3)	36	0.4
7	Platelet	150-450 ×103/μL	267.3 (143.9)	118	239.0 (115.0)	34	0.3
8	Urea	15-40mg/dl	30.9 (9.2)	121	33.8 (12)	36	0.1
9	Creatinine	0.6-1.2mg/dL	0.9 (2.7)	118	0.7 (0.3)	36	0.6
10	Estimated GFR	>60mL/min/1.73m2	92.1 (17.8)	117	91.7 (22.9)	36	0.9
11	Albumin	3.4-5.0g/dL	3.9 (0.5)	119	3.8 (0.5)	37	0.5
12	Bilirubin	0.3-1.2mg/dL	0.7 (0.4)	120	0.8 (1.2)	37	0.4
13	AST	20-45U/L	29 (12.8)	121	27.5 (8.5)	37	0.5
14	ALT	10-40U/L	24.5 (16)	121	24.4 (11.7)	37	1.0
15	Sodium	136-142mEq/L	137.7 (12.7)	122	139.2 (6.2)	37	0.5
16	Potassium	3.5-5.0mEq/L	4.6 (0.5)	121	4.6 (0.5)	37	0.7
17	Phosphate	2.3-4.7mg/dL	3.8 (0.9)	123	4 (0.9)	36	0.6
18	Calcium	8.2-10.2mg/dL	9.8 (0.8)	122	9.7 (0.8)	37	0.4
19	Corrected calcium	8.2-10.2mg/dL	9.9 (0.8)	122	9.8 (0.7)	37	0.5
20	PTH	10-66pg/mL	31.6 (21)	108	29.2 (17.8)	39	0.5
21	Vitamin D	20-80ng/mL	44 (26.3)	106	48.5 (29.6)	38	0.4
22	ALP	50-129U/L	102.9 (37.1)	122	90.8 (29.3)	36	0.07

Abbreviations - N: Number of patients whose data were available, n: total number of subjects in the group. ALT: Alanine aminotransferase, ALP: Alkaline phosphatase, AST: Aspartate aminotransferase, GFR: Glomerular filtration rate, PTH: Parathyroid hormone, SD: Standard deviation, TLC: Total leucocyte count.

**Table IV:** Relative prevalence of selected crucial laboratory abnormalities calculated using regression analysis. "corrected calcium = serum calcium + 0.8 (4 - serum albumin)", taking 4gm/dl albumin as the reference value

Variable	Group-L n/N (%)	Group-H n/N (%)	PR (95% CI)	P-value
Hypercalcemia (>10.4mg/dl)	23/122 (18.9)	6/37 (16.2%)	1.2 (0.4-3.2)	0.72
Hyperphosphatemia (>4.7mg/dl)	11/123 (8.9%)	4/36 (11.1%)	0.8 (0.2-2.6)	0.70
Hyperparathyroidism (>66pg/ml)	9/108 (8.3%)	2/39 (5.1%)	1.7 (0.3-8.1)	0.73
Vitamin D deficiency (<20ng/ml)	18/106 (17.0%)	7/38 (18.4%)	0.9 (0.3-2.4)	0.84
eGFRcr <60	7/117 (6.0 %)	5/36 (13.9%)	0.4 (0.1-1.3)	0.12
Raised ALP (>129IU/L)	19/122 (15.6%)	2/36 (5.6%)	3.1 (0.7-14.1)	0.16
Hypoalbuminemia (<3.4gm/dl)	19/119 (16.0%)	8/37 (21.6%)	0.7 (0.3-1.7)	0.43
Raised AST (>40IU/L)	13/121 (10.7%)	2/37 (5.4%)	2.1 (0.5-9.8)	0.34
Raised ALT (>45IU/L)	10/121 (8.3%)	3/37 (8.1%)	1.0 (0.3-3.9)	1.0
Anaemia (<10gm/dl)	14/120 (11.7%)	6/37 (16.2%)	0.7 (0.2-1.9)	0.47
Abnormal Sodium (>148 or <135mmol/L)	28/122 (23.0%)	9/37 (24.3%)	0.9 (0.4-2.2)	0.86
Abnormal Potassium (>5 or <3.5mmol/L)	24/121 (19.8%)	7/37 (18.9%)	1.0 (0.4-2.7)	0.90
At least one selected parameter	89/124 (71.8%)	30/49 (61.2%)	1.6 (0.8-3.2)	0.18

Abbreviations - n: number of patients qualifying the criteria, N: number of patients whose data were available. ALT: Alanine aminotransferase, ALP: Alkaline phosphatase, AST: Aspartate aminotransferase, CI: Confidence interval, eGFRcr: Estimated glomerular filtration rate based on the serum creatinine, PR: Prevalence ratio.

## Discussion

Due to the recent surge in awareness regarding fragility fractures, patients older than 50 years suffering a fracture following trivial trauma undergo extensive clinical, radiological, and laboratory workup routinely^6-9^. This protocol diagnoses underlying comorbidities at an earlier stage and improves the overall prognosis of the patient. However, there is a lack of such similar protocols in literature for older patients suffering high energy trauma. This lacunae in literature made the basis for the present study.

The routine laboratory works up for fragility fractures include BMD, Liver function tests, Kidney functions tests, serum calcium, phosphorus, alkaline phosphatase and vitamin D levels^6-9^. We will now discuss the role of each factor in the management of high and low energy fractures in older patients. Many authors have noticed that only a few lab tests are associated with reduced BMD^17,18^. However, the cause of fragility fracture is multifactorial, and its risk depends on many factors other than the BMD^4^. Therefore, BMD is specific but is less sensitive in estimating the risk for secondary fractures. Even in our study, 43.5% of patients in the fragility fracture group had a BMD of more than -2.5. This finding is supported by previous literature^4,19,20^. Malgo *et al* found that 3/4th of the patient who had sustained a fragility fracture had a BMD >-2.519. Furthermore, a level 1 study by Siris and colleagues suggested that most fragility fractures will happen in women who are non-osteoporotic as per the world health organisation definition, with a T-score more than -2.520. The NICE guidelines also do not recommend the use of BMD testing alone for population screening4. Therefore, an extensive routine laboratory analysis of such patients is recommended, for fragility fracture workup^6-9^.

In the current study, among fractures present in at least 3% of patients of either group, distal radius fracture was significantly more common in group-L, while proximal tibia fracture was significantly more common among group-H patients (Table I). This finding is concurrent with those in the literature wherein 90% of patients older than 50 years with distal radius fracture had a low energy trauma^21,22^. In the high energy group, thirteen out of 49 patients (24%) had a pertrochanteric fracture. The authors agree that this percentage is comparatively higher than reported in the literature^23^. This discrepancy is probably due to a liberal inclusion criterion of a fall from standing height to be considered a high energy trauma. None of the patients in the high energy group had a fracture of the pelvis or acetabulum. This finding might be explained by the fact that we have excluded patients with multiple injuries and systemic complications, which these patients usually have suffered.

In our study, hypoalbuminemia was present in more than one-fifth of patients with high energy trauma and was the most common derangement among liver function tests. Chronic liver diseases negatively influence bone quality. Patients with compromised liver function have been shown to suffer from a worse outcome following hip fractures with increased hospitalisation, poor wound healing, increased chances of infection, and elevated in-hospital mortality rate^24,25^. Moreover, in a population-based survey, elevated liver enzymes were inversely related to the BMD^26^. These patients also have an increased risk of bleeding which further compromise the outcome^24^. The high prevalence of hepatic dysfunction among older patients with high energy injuries justifies the need to carry out liver function tests routinely in them.

In the present study, five patients of the high energy group and 6% of patients with fragility fracture were having stage three to five CKD (eGFRcr less than 60mL/min/1.73m^2^) (P=0.12). Stage two CKD (eGFRcr 60- 90mL/min/1.73m^2^) was present in seven patients of group-H and 30.8% patients of group-L. Previous literature suggests that even stage two CKD is associated with reduced BMD and an increased risk of vertebral fractures^13^. A comprehensive population-based survey revealed that patients with eGFR less than 60mL/min/1.73m^2^ suffer more frequently from a hip fracture and have a high mortality rate^27^. Thus, if we estimate the eGFRcr in every older patient with a fracture, we can diagnose even mild renal dysfunction and improve the overall outcome.

Patients with any derangement in indices of calcium metabolism need proper management to decrease the risk of future fractures and improve the outcome^5,28,29^. In the current study, calcium metabolism was abnormal in equal proportion in both groups. Similar results were also reported by Lee *et al*, who have compared the parameters of calcium metabolism and bone turnover markers of post-menopausal women based on the severity of the trauma and have noticed no significant difference among them^30^. In our study, 11 patients had hyperparathyroidism. Among these patients, five had either CKD or vitamin D insufficiency, five had normocalcemic hyperparathyroidism, and one had primary hyperparathyroidism. Furthermore, twenty-nine patients of our study had hypercalcemia, the cause of which was either renal-failure or hyperparathyroidism in five patients, while the rest 24 patients required further evaluation for the determination of the primary ailment. Therefore, the authors emphasise the need to carry out these investigations in all older patients with a fracture, including those with high energy injury.

We have identified overall Vitamin D deficiency and insufficiency (levels <30ng/ml) in around one-third of our cases across both groups. Previous reports have also reported a high incidence of vitamin D insufficiency in the urban elderly population^31^. Literature suggests prescribing vitamin D supplements to every patient with a fragility fracture, particularly the elderly patients with a hip fracture^32^. Some researchers disapprove of the need for routine Vitamin D analysis as its deficiency is almost universal^14^. However, a recent meta-analysis doesn’t recommend vitamin D supplementation to every trauma patient older than 50 years who live in their communities^33^. Furthermore, Vitamin D levels are also essential in identifying the cause of deranged calcium and parathyroid hormone levels. Therefore, we recommend checking Vitamin D levels in all older patients with a fracture.

An out-of-range serum sodium level was the most common laboratory irregularity in our study, which was present in 23% of the subjects across both groups. Among these patients, hyponatremia was present in 54%. The latter has been implicated as an independent risk factor of fractures^34^. It not only increases the risk of falls but also reduces the quality of bone^35^. Thus, correcting this ailment would help us in reducing the risk of future fractures and improve the survival rate of these patients. Therefore, the authors justify conducting serum electrolytes screening in all older patients regardless of the mechanism of injury.

The average haemoglobin levels of both groups were slightly lower than the normal range. Since we have excluded patients with systemic complications of trauma which usually involve patients with multiple fractures and with massive blood loss, the patients with anaemia are lesser in our groups. Furthermore, we have taken the follow-up investigation into account which represent the baseline status of the patients; thus, the effect of trauma must have been weaned off. Moderate or severe anaemia, which is associated with poor prognosis following a fracture^36^, was present in six patients in group-H and 11.7% patients of group-L (P=0.47). Thus, performing a haematological analysis is essential in patients with high energy injury as in fragility fractures.

In the current study, almost three out of four subjects with a fragility fracture and more than three-fifths of the patients with severe trauma had at least one laboratory abnormality (P=0.18). Furthermore, the ROC curve showed that the patient's chance of having more abnormal laboratory tests were independent of the mechanism of injury. These findings further establish the need for performing a detailed laboratory analysis in all older patients with a fracture.

Ours is the first study that does a comprehensive biochemical analysis in high energy trauma patients of the older age group to show the importance of such investigations in them. Our study has certain limitations as listed below. The single-centre cross-sectional study design has its drawbacks. Second, we have not analysed the effect of factors such as alcohol consumption, smoking, drug history, hormonal analysis, and dietary calcium intake on our results. Finally, the traumatic fracture group had a relatively less number of subjects, with a higher proportion of female subjects and more missing data. Future studies with an ideal sample size will help in better assessment of these relationships.

## Conclusion

Our results showed that the frequency of comorbidities in the high energy group was similar to the low energy group. Thus, proving our hypothesis that high energy group of patients need a dedicated fracture liaison service to detect their comorbidities on similar lines as the low energy group of patients. Furthermore, older patients with high energy trauma would benefit equally from a comprehensive laboratory workup during their follow-up visit as the fragility fracture patients, which would help improve their prognosis concerning their long-term survival and functional outcomes.
